# fqtools: an efficient software suite for modern FASTQ file manipulation

**DOI:** 10.1093/bioinformatics/btw088

**Published:** 2016-02-18

**Authors:** Alastair P. Droop

**Affiliations:** MRC Medical Bioinformatics Centre, University of Leeds, Clarendon Way, Leeds, LS2 9NL, UK

## Abstract

**Summary:** Many Next Generation Sequencing analyses involve the basic manipulation of input sequence data before downstream processing (e.g. searching for specific sequences, format conversion or basic file statistics). The rapidly increasing data volumes involved in NGS make any dataset manipulation a time-consuming and error-prone process. I have developed fqtools; a fast and reliable FASTQ file manipulation suite that can process the full set of valid FASTQ files, including those with multi-line sequences, whilst identifying invalid files. Fqtools is faster than similar tools, and is designed for use in automatic processing pipelines.

**Availability and implementation:** fqtools is open source and is available at: https://github.com/alastair-droop/fqtools.

**Supplementary information**: Supplementary data are available at *Bioinformatics* online.

**Contact:** a.p.droop@leeds.ac.uk

## 1 Introduction

The FASTQ format has become the *de facto* standard for storage of next-generation sequencing read data (Cock *et al.*, 2010). Based originally upon the FASTA sequence format (Pearson and Lipman, 1988), FASTQ stores nucleotide sequences and associated base qualities (Ewing and Green, 1998) for multiple named reads in a four-field human-readable ASCII format. Although there is no defined standard for FASTQ files, Cock *et al.* (2010) provide a good overview of the format, and provide as close to a ‘standard’ as is available.

Many analysis pipelines involve initial data manipulation (e.g. reformatting, viewing or overview statistics) before downstream processing (e.g. quality control, adapter removal and alignment). Seemingly simple tasks like viewing the first few reads in a file or checking the distribution of read lengths often require scripting or loading the data in tools that are quite slow for large datasets. These file manipulations are much more frequent when data are being re-used in novel analyses. Frequently, individual researchers will write scripts (e.g. in Python, Perl or AWK) to perform these tasks. Many tools are available for FASTQ processing such as the fastx-toolkit, bio-awk, fastq-tools, fast, seqmagick and seq-tk (see the Supplementary Materials for the URLs of these tools). None of these provide a comprehensive set of common manipulations that would be required for most analyses.

Most FASTQ processing tools fail to process reads with sequence data split across multiple lines. As read lengths from modern sequencing technologies are constantly increasing (Schneider and Dekker, 2012), this is likely to become problematic as human readability is vastly reduced by extremely long lines.

Detecting invalid input is extremely important, as bioinformatics pipelines are often automated; thus significant computation and analysis time can be wasted if input errors are not detected early. For this reason, a trustworthy FASTQ manipulation tool should report invalid files. Similarly, tools should be able to correctly process the full range of valid inputs.

The fqtools suite was written to address this need for efficient and reliable viewing, manipulation and summarization of FASTQ data before it is pre-processed (with e.g. FastQC [http://www.bioinformatics.babraham.ac.uk/projects/fastqc/) or cutadapt (Martin, 2011)]. Both compressed and plain FASTQ can be processed, as can SAM and BAM-formatted data. Paired-end sequence data is handled either as file pairs or in interleaved format (Table 1).

**Table 1.** Commands present in the fqtools suite

	Description
view	View FASTQ files
head	View the first reads in FASTQ files
count	Count FASTQ file reads
header	View FASTQ file header data
sequence	View FASTQ file sequence data
quality	View FASTQ file quality data
header2	View FASTQ file secondary header data
fasta	Convert FASTQ files to FASTA format
basetab	Tabulate FASTQ base frequencies
qualtab	Tabulate FASTQ quality character frequencies
lengthtab	Tabulate FASTQ read lengths
type	Attempt to guess the FASTQ quality encoding type
validate	Validate FASTQ files
find	Find FASTQ reads containing specific sequences
trim	Trim reads in a FASTQ file
qualmap	Translate quality values using a mapping file

Commands present in the fqtools suite. The supplementary information contains a full description of each command.

## 2 Implementation

I have developed a fast and memory-efficient state machine for parsing FASTQ files. The use of a state machine (as opposed to a line-based approach) obviates the difficulties with line breaks in sequence and quality data. As read order must be identical for both paired-end files, manipulations that re-order reads process both pairs simultaneously.

The fqtools suite has been written to allow input and output from either files or standard streams. Both files and streams can contain either plain or gzip-compressed data. By using streams, the fqtools suite can be easily incorporated into computational pipelines.

The commands contained in the fqtools suite are listed in Table 2, along with a brief description of their purpose.

**Table 2.** FASTQ processing tools overview

	Valid	Invalid	Process .gz	Plain (reads/s)	Compressed (reads/)
fqtools	Y	Y	R+W	701 375	444 648
bash	—	—	R+W	2 605 421	934 331
bioawk	Y	N	R	434 632	312 708
seqtk	Y	N	R	1 122 355	545 865
fast	Y	Y	—	2984	—
fastx-toolkit	N	N	—	69 762	—
seqmagick	Y	Y	R+W	25 325	4000

Benchmark data for various FASTQ processing tools. All tools were installed locally, and run against the complete test set (Cock *et al*., 2010). Valid shows if all the valid test set were processed correctly. Invalid shows if the tool identified all the invalid files. Process .gz shows if the tool can natively read (R) and write (W) gzip-compressed files. The speed columns show the speed in reads per second.

## 3 Performance

I tested several common sequence manipulation tools against four criteria:

- The ability to process the full range of valid FASTQ files;
- The ability to detect the full range of FASTQ errors;
- The ability to read and write compressed data; and
- The processing speed.

To evaluate the ability of the fqtools suite to correctly process valid files and to reject invalid ones, I used the test set provided by Cock *et al.* (2010). The performance of the fqtools suite was tested against several similar tools using a sample file containing 100 000 reads generated using ART (Huang *et al.*, 2012). Table 2 shows these results. For all tools, the closest option to parsing the file without further processing was used. The lowest time score over 50 repeats was taken. Speed results for printing the files on the bash terminal are supplied for a ‘maximum speed’ reference, although these commands make no attempt to parse the FASTQ data within the file. Full data are available in the supplementary information.

Of the tools tested, only three (fqtools, fast and seqmagick) correctly processed the full test set. Of these three, fqtools is by far the fastest when processing both uncompressed and compressed files.

Although several of these tools are designed to be extendable, data processing speed for basic FASTQ manipulation is becoming increasingly important. For these tasks, the flexibility of interpreted tools (such as seqmagick and fast) is unnecessary.

## 4 Summary

Here, I describe fastqc, a suite of FASTQ manipulations tools that efficiently handles the full range of valid FASTQ, whilst detecting invalid files. The suite can process both compressed and uncompressed files. fqtools is freely available on Github at https://github.com/alastair-droop/fqtools.

## Supplementary Material

Supplementary Data
