# Supplementary material for: fqtools: an efficient software suite for modern FASTQ file manipulation
Source: Bioinformatics. 2016 Feb 18;32(12):1883–4. doi: 10.1093/bioinformatics/btw088 (PMC4908325; doi:10.1093/bioinformatics/btw088)
Supplement: Supplementary Data [file supp_32_12_1883__index.html]

fqtools: an efficient software suite for modern FASTQ file manipulation — fqtools: an efficient software suite for modern FASTQ file manipulation — Supplementary Data 

# fqtools: an efficient software suite for modern FASTQ file manipulation

## Supplementary Data

files

- Supplementary Data - pdf file
