# Supplementary material for: fqtools: an efficient software suite for modern FASTQ file manipulation
Source: Bioinformatics. 2016 Feb 18;32(12):1883–4. doi: 10.1093/bioinformatics/btw088 (PMC4908325; doi:10.1093/bioinformatics/btw088)
Supplement: Supplementary Data [file supp_btw088_fqtools-supplementary-data.pdf]

fqtools: An efficient software suite for modern FASTQ file manipulation – Supplementary data

Tool URLs

fqtools	https://github.com/alastair-droop/fqtools
fastx-toolkit	http://hannonlab.cshl.edu/fastx_toolkit/
bio-awk	https://github.com/lh3/bioawk
fastq-tools	http://homes.cs.washington.edu/~dcjones/fastq-tools/
fast	https://github.com/tlawrence3/FAST
seqmagick	https://github.com/fhcrc/seqmagick
seq-tk	https://github.com/lh3/seqtk

FQTools Commands

The following commands are included in the fqtools suite (version ≥2.0):

view

This command simply prints the contents of the file or file pair back in a standard format. Thus, all line breaks are removed. This is an analogue of the unix `cat` command.

head

This command is the same as view, but will only return the first few reads of a file. This is an analogue of the unix `head` command.

count

This command counts the number of reads present in a FASTQ file (or pair of files).

header

This command returns only the primary header data for all reads in a FASTQ file. If paired data are given, the output will contain two columns.

sequence

This command returns only the sequence data for all reads in a FASTQ file. If paired data are given, the output will contain two columns.

quality

This command returns only the quality data for all reads in a FASTQ file. If paired data are given, the output will contain two columns.

header2

This command returns only the secondary headerdata for all reads in a FASTQ file. If paired data are given, the output will contain two columns.

fasta

This command returns all reads in FASTA format (thus stripping quality data).

basetab

This command tabulates the nucleotide base frequencies present in the input data (either across a single file or both files in a pair of files).

qualtab

This command tabulates the quality character frequencies across a single FASTQ file or pair of files. If a quality encoding strategy is specified, it will also show the score and associat6ed error probabilities.

lengthtab

This command tabulates the read lengths across all reads in a FASTQ fiel or pair of files.

type

This command will attempt to guess the quality encoding type of a single FASTQ file based upon its quality character distribution.

validate

This command will validate a FASTQ file or pair of files, checking for validity.

find

This command will find specific nucleotide sequences, returning only those reads containing the subsequence.

trim

This command trims reads. This is simple trimming (removal of initial fixed length regions, or by total length).

qualmap

This command allows re-mapping of quality scores. A translation table is specified and all quality scores are translated accordingly. This is very useful when binning quality data to fit with Illumina's new quality binning strategies.

Validity Testing

Testing for a tool's validity was performed against the cock *et al.* dataset (Cock *et al.*, 2010). Each tool was run against each file using Python. The commands used are shown in table S1. The individual file results are shown in table S2 (below).

Table S1: The commands used for validity testing. The environment variable `$FQFILE` is the current FASTQ file to test.

Tool	Command
**fast**	`faslen -q -t $FQFILE`
**fqtools**	`fqtools -dramul validate $FQFILE`
**seqtk**	`seqtk seq -l0 $FQFILE`
**bioawk**	`bioawk -cfastx '{{print "@"$name; print $seq; print "+"; print $qual}}' $FQFILE`
**seqmagick**	`seqmagick info $FQFILE`
**fastx-toolkit**	`fastx_quality_stats -i $FQFILE`

Speed Testing

As not all tools can perform all analyses, the command closest to simply parsing a file without any further processing was chosen. The commands selected are shown in table S3. For the speed testing, a random FASTQ was generated using ART (Huang *et al.*, 2012) containing 100,000 reads all of which have 150 cycles. Speed testing of compressed data used the same file after gzip compression. For each tool, the fastest run time over several runs (n=50) on a standard machine was recorded.

Table S1: The commands used for speed testing. The environment variable `$IN` is the current FASTQ file to test; `$OUT` is the resulting output from the tool.

Tool	Command
**bash-compressed**	`gzcat  $IN > $OUT`
**bash-plain**	`cat  $IN > $OUT`
**bioawk**	`bioawk -cfastx '{{print "@"$name; print $seq; print "+"; print $qual}}' $IN > $OUT`
**fast**	`fashead -q -n100000 $IN > $OUT`
**fastx-toolkit**	`fastx_renamer -nSEQ -i$IN > $OUT`
**fqtools**	`fqtools view $IN > $OUT`
**seqmagick**	`seqmagick convert $IN $OUT`
**seqtk**	`seqtk seq $IN > $OUT`

References

Cock,P.J.A. *et al.* (2010) The Sanger FASTQ file format for sequences with quality scores, and the Solexa/Illumina FASTQ variants. *Nucleic Acids Research*, **38**, 1767–1771.

Huang,W. *et al.* (2012) ART: a next-generation sequencing read simulator. *Bioinformatics*, **28**, 593–594.

Table S2: Individual file results for validity testing. Each command was run against all the FASTQ files, and the error status reported.

Tool	File status	File	Result
**fastx-toolkit**	valid	illumina_full_range_as_illumina	PSS
**fastx-toolkit**	valid	sanger_full_range_as_illumina	PASS
**fastx-toolkit**	valid	longreads_as_illumina	FAIL
**fastx-toolkit**	valid	solexa_full_range_as_illumina	PASS
**fastx-toolkit**	valid	sanger_full_range_as_sanger	PASS
**fastx-toolkit**	valid	longreads_as_sanger	FAIL
**fastx-toolkit**	valid	solexa_full_range_as_solexa	PASS
**fastx-toolkit**	valid	misc_dna_original_sanger	FAIL
**fastx-toolkit**	valid	misc_dna_as_sanger	FAIL
**fastx-toolkit**	valid	misc_rna_original_sanger	FAIL
**fastx-toolkit**	valid	misc_rna_as_illumina	FAIL
**fastx-toolkit**	valid	misc_rna_as_sanger	FAIL
**fastx-toolkit**	valid	sanger_full_range_original_sanger	PASS
**fastx-toolkit**	valid	wrapping_as_solexa	PASS
**fastx-toolkit**	valid	illumina_full_range_as_sanger	PASS
**fastx-toolkit**	valid	illumina_full_range_original_illumina	PASS
**fastx-toolkit**	valid	longreads_as_solexa	FAIL
**fastx-toolkit**	valid	longreads_original_sanger	FAIL
**fastx-toolkit**	valid	misc_dna_as_solexa	FAIL
**fastx-toolkit**	valid	solexa_full_range_original_solexa	PASS
**fastx-toolkit**	valid	misc_rna_as_solexa	FAIL
**fastx-toolkit**	valid	wrapping_original_sanger	FAIL
**fastx-toolkit**	valid	solexa_full_range_as_sanger	PASS
**fastx-toolkit**	valid	wrapping_as_illumina	PASS
**fastx-toolkit**	valid	wrapping_as_sanger	PASS
**fastx-toolkit**	valid	illumina_full_range_as_solexa	PASS
**fastx-toolkit**	valid	misc_dna_as_illumina	FAIL
**fastx-toolkit**	valid	sanger_full_range_as_solexa	PASS
**fastx-toolkit**	invalid	error_qual_del	PASS
**fastx-toolkit**	invalid	error_diff_ids	FAIL
**fastx-toolkit**	invalid	error_qual_null	PASS
**fastx-toolkit**	invalid	error_trunc_at_seq	PASS
**fastx-toolkit**	invalid	error_qual_unit_sep	FAIL
**fastx-toolkit**	invalid	error_double_seq	PASS
**fastx-toolkit**	invalid	error_trunc_at_qual	PASS
**fastx-toolkit**	invalid	error_trunc_in_qual	PASS
**fastx-toolkit**	invalid	error_qual_escape	FAIL
**fastx-toolkit**	invalid	error_trunc_at_plus	PASS
**fastx-toolkit**	invalid	error_no_qual	PASS
**fastx-toolkit**	invalid	error_qual_tab	PASS
**fastx-toolkit**	invalid	error_trunc_in_seq	PASS

**fastx-toolkit**	invalid	error_qual_vtab	PASS
**fastx-toolkit**	invalid	error_qual_space	PASS
**fastx-toolkit**	invalid	error_long_qual	PASS
**fastx-toolkit**	invalid	error_trunc_in_title	PASS
**fastx-toolkit**	invalid	error_trunc_in_plus	PASS
**fastx-toolkit**	invalid	error_short_qual	PASS
**fastx-toolkit**	invalid	error_tabs	PASS
**fastx-toolkit**	invalid	error_spaces	PASS
**fastx-toolkit**	invalid	error_double_qual	PASS
**seqtk**	valid	illumina_full_range_as_illumina	PASS
**seqtk**	valid	sanger_full_range_as_illumina	PASS
**seqtk**	valid	longreads_as_illumina	PASS
**seqtk**	valid	solexa_full_range_as_illumina	PASS
**seqtk**	valid	sanger_full_range_as_sanger	PASS
**seqtk**	valid	longreads_as_sanger	PASS
**seqtk**	valid	solexa_full_range_as_solexa	PASS
**seqtk**	valid	misc_dna_original_sanger	PASS
**seqtk**	valid	misc_dna_as_sanger	PASS
**seqtk**	valid	misc_rna_original_sanger	PASS
**seqtk**	valid	misc_rna_as_illumina	PASS
**seqtk**	valid	misc_rna_as_sanger	PASS
**seqtk**	valid	sanger_full_range_original_sanger	PASS
**seqtk**	valid	wrapping_as_solexa	PASS
**seqtk**	valid	illumina_full_range_as_sanger	PASS
**seqtk**	valid	illumina_full_range_original_illumina	PASS
**seqtk**	valid	longreads_as_solexa	PASS
**seqtk**	valid	longreads_original_sanger	PASS
**seqtk**	valid	misc_dna_as_solexa	PASS
**seqtk**	valid	solexa_full_range_original_solexa	PASS
**seqtk**	valid	misc_rna_as_solexa	PASS
**seqtk**	valid	wrapping_original_sanger	PASS
**seqtk**	valid	solexa_full_range_as_sanger	PASS
**seqtk**	valid	wrapping_as_illumina	PASS
**seqtk**	valid	wrapping_as_sanger	PASS
**seqtk**	valid	illumina_full_range_as_solexa	PASS
**seqtk**	valid	misc_dna_as_illumina	PASS
**seqtk**	valid	sanger_full_range_as_solexa	PASS
**seqtk**	invalid	error_qual_del	FAIL
**seqtk**	invalid	error_diff_ids	FAIL
**seqtk**	invalid	error_qual_null	FAIL
**seqtk**	invalid	error_trunc_at_seq	FAIL
**seqtk**	invalid	error_qual_unit_sep	FAIL
**seqtk**	invalid	error_double_seq	FAIL
**seqtk**	invalid	error_trunc_at_qual	FAIL
**seqtk**	invalid	error_trunc_in_qual	FAIL
**seqtk**	invalid	error_qual_escape	FAIL

**seqtk**	invalid	error_trunc_at_plus	FAIL
**seqtk**	invalid	error_no_qual	FAIL
**seqtk**	invalid	error_qual_tab	FAIL
**seqtk**	invalid	error_trunc_in_seq	FAIL
**seqtk**	invalid	error_qual_vtab	FAIL
**seqtk**	invalid	error_qual_space	FAIL
**seqtk**	invalid	error_long_qual	FAIL
**seqtk**	invalid	error_trunc_in_title	FAIL
**seqtk**	invalid	error_trunc_in_plus	FAIL
**seqtk**	invalid	error_short_qual	FAIL
**seqtk**	invalid	error_tabs	FAIL
**seqtk**	invalid	error_spaces	FAIL
**seqtk**	invalid	error_double_qual	FAIL
**bioawk**	valid	illumina_full_range_as_illumina	PASS
**bioawk**	valid	sanger_full_range_as_illumina	PASS
**bioawk**	valid	longreads_as_illumina	PASS
**bioawk**	valid	solexa_full_range_as_illumina	PASS
**bioawk**	valid	sanger_full_range_as_sanger	PASS
**bioawk**	valid	longreads_as_sanger	PASS
**bioawk**	valid	solexa_full_range_as_solexa	PASS
**bioawk**	valid	misc_dna_original_sanger	PASS
**bioawk**	valid	misc_dna_as_sanger	PASS
**bioawk**	valid	misc_rna_original_sanger	PASS
**bioawk**	valid	misc_rna_as_illumina	PASS
**bioawk**	valid	misc_rna_as_sanger	PASS
**bioawk**	valid	sanger_full_range_original_sanger	PASS
**bioawk**	valid	wrapping_as_solexa	PASS
**bioawk**	valid	illumina_full_range_as_sanger	PASS
**bioawk**	valid	illumina_full_range_original_illumina	PASS
**bioawk**	valid	longreads_as_solexa	PASS
**bioawk**	valid	longreads_original_sanger	PASS
**bioawk**	valid	misc_dna_as_solexa	PASS
**bioawk**	valid	solexa_full_range_original_solexa	PASS
**bioawk**	valid	misc_rna_as_solexa	PASS
**bioawk**	valid	wrapping_original_sanger	PASS
**bioawk**	valid	solexa_full_range_as_sanger	PASS
**bioawk**	valid	wrapping_as_illumina	PASS
**bioawk**	valid	wrapping_as_sanger	PASS
**bioawk**	valid	illumina_full_range_as_solexa	PASS
**bioawk**	valid	misc_dna_as_illumina	PASS
**bioawk**	valid	sanger_full_range_as_solexa	PASS
**bioawk**	invalid	error_qual_del	FAIL
**bioawk**	invalid	error_diff_ids	FAIL
**bioawk**	invalid	error_qual_null	FAIL
**bioawk**	invalid	error_trunc_at_seq	FAIL
**bioawk**	invalid	error_qual_unit_sep	FAIL

**bioawk**	invalid	error_double_seq	FAIL
**bioawk**	invalid	error_trunc_at_qual	FAIL
**bioawk**	invalid	error_trunc_in_qual	FAIL
**bioawk**	invalid	error_qual_escape	FAIL
**bioawk**	invalid	error_trunc_at_plus	FAIL
**bioawk**	invalid	error_no_qual	PASS
**bioawk**	invalid	error_qual_tab	FAIL
**bioawk**	invalid	error_trunc_in_seq	FAIL
**bioawk**	invalid	error_qual_vtab	FAIL
**bioawk**	invalid	error_qual_space	FAIL
**bioawk**	invalid	error_long_qual	FAIL
**bioawk**	invalid	error_trunc_in_title	FAIL
**bioawk**	invalid	error_trunc_in_plus	FAIL
**bioawk**	invalid	error_short_qual	FAIL
**bioawk**	invalid	error_tabs	FAIL
**bioawk**	invalid	error_spaces	FAIL
**bioawk**	invalid	error_double_qual	FAIL
**seqmagick**	valid	illumina_full_range_as_illumina	PASS
**seqmagick**	valid	sanger_full_range_as_illumina	PASS
**seqmagick**	valid	longreads_as_illumina	PASS
**seqmagick**	valid	solexa_full_range_as_illumina	PASS
**seqmagick**	valid	sanger_full_range_as_sanger	PASS
**seqmagick**	valid	longreads_as_sanger	PASS
**seqmagick**	valid	solexa_full_range_as_solexa	PASS
**seqmagick**	valid	misc_dna_original_sanger	PASS
**seqmagick**	valid	misc_dna_as_sanger	PASS
**seqmagick**	valid	misc_rna_original_sanger	PASS
**seqmagick**	valid	misc_rna_as_illumina	PASS
**seqmagick**	valid	misc_rna_as_sanger	PASS
**seqmagick**	valid	sanger_full_range_original_sanger	PASS
**seqmagick**	valid	wrapping_as_solexa	PASS
**seqmagick**	valid	illumina_full_range_as_sanger	PASS
**seqmagick**	valid	illumina_full_range_original_illumina	PASS
**seqmagick**	valid	longreads_as_solexa	PASS
**seqmagick**	valid	longreads_original_sanger	PASS
**seqmagick**	valid	misc_dna_as_solexa	PASS
**seqmagick**	valid	solexa_full_range_original_solexa	PASS
**seqmagick**	valid	misc_rna_as_solexa	PASS
**seqmagick**	valid	wrapping_original_sanger	PASS
**seqmagick**	valid	solexa_full_range_as_sanger	PASS
**seqmagick**	valid	wrapping_as_illumina	PASS
**seqmagick**	valid	wrapping_as_sanger	PASS
**seqmagick**	valid	illumina_full_range_as_solexa	PASS
**seqmagick**	valid	misc_dna_as_illumina	PASS
**seqmagick**	valid	sanger_full_range_as_solexa	PASS
**seqmagick**	invalid	error_qual_del	PASS

**seqmagick**	invalid	error_diff_ids	PASS
**seqmagick**	invalid	error_qual_null	PASS
**seqmagick**	invalid	error_trunc_at_seq	PASS
**seqmagick**	invalid	error_qual_unit_sep	PASS
**seqmagick**	invalid	error_double_seq	PASS
**seqmagick**	invalid	error_trunc_at_qual	PASS
**seqmagick**	invalid	error_trunc_in_qual	PASS
**seqmagick**	invalid	error_qual_escape	PASS
**seqmagick**	invalid	error_trunc_at_plus	PASS
**seqmagick**	invalid	error_no_qual	PASS
**seqmagick**	invalid	error_qual_tab	PASS
**seqmagick**	invalid	error_trunc_in_seq	PASS
**seqmagick**	invalid	error_qual_vtab	PASS
**seqmagick**	invalid	error_qual_space	PASS
**seqmagick**	invalid	error_long_qual	PASS
**seqmagick**	invalid	error_trunc_in_title	PASS
**seqmagick**	invalid	error_trunc_in_plus	PASS
**seqmagick**	invalid	error_short_qual	PASS
**seqmagick**	invalid	error_tabs	PASS
**seqmagick**	invalid	error_spaces	PASS
**seqmagick**	invalid	error_double_qual	PASS
**fast**	valid	illumina_full_range_as_illumina	PASS
**fast**	valid	sanger_full_range_as_illumina	PASS
**fast**	valid	longreads_as_illumina	PASS
**fast**	valid	solexa_full_range_as_illumina	PASS
**fast**	valid	sanger_full_range_as_sanger	PASS
**fast**	valid	longreads_as_sanger	PASS
**fast**	valid	solexa_full_range_as_solexa	PASS
**fast**	valid	misc_dna_original_sanger	PASS
**fast**	valid	misc_dna_as_sanger	PASS
**fast**	valid	misc_rna_original_sanger	PASS
**fast**	valid	misc_rna_as_illumina	PASS
**fast**	valid	misc_rna_as_sanger	PASS
**fast**	valid	sanger_full_range_original_sanger	PASS
**fast**	valid	wrapping_as_solexa	PASS
**fast**	valid	illumina_full_range_as_sanger	PASS
**fast**	valid	illumina_full_range_original_illumina	PASS
**fast**	valid	longreads_as_solexa	PASS
**fast**	valid	longreads_original_sanger	PASS
**fast**	valid	misc_dna_as_solexa	PASS
**fast**	valid	solexa_full_range_original_solexa	PASS
**fast**	valid	misc_rna_as_solexa	PASS
**fast**	valid	wrapping_original_sanger	PASS
**fast**	valid	solexa_full_range_as_sanger	PASS
**fast**	valid	wrapping_as_illumina	PASS
**fast**	valid	wrapping_as_sanger	PASS

**fast**	valid	illumina_full_range_as_solexa	PASS
**fast**	valid	misc_dna_as_illumina	PASS
**fast**	valid	sanger_full_range_as_solexa	PASS
**fast**	invalid	error_qual_del	PASS
**fast**	invalid	error_diff_ids	PASS
**fast**	invalid	error_qual_null	PASS
**fast**	invalid	error_trunc_at_seq	PASS
**fast**	invalid	error_qual_unit_sep	PASS
**fast**	invalid	error_double_seq	PASS
**fast**	invalid	error_trunc_at_qual	PASS
**fast**	invalid	error_trunc_in_qual	PASS
**fast**	invalid	error_qual_escape	PASS
**fast**	invalid	error_trunc_at_plus	PASS
**fast**	invalid	error_no_qual	PASS
**fast**	invalid	error_qual_tab	PASS
**fast**	invalid	error_trunc_in_seq	PASS
**fast**	invalid	error_qual_vtab	PASS
**fast**	invalid	error_qual_space	PASS
**fast**	invalid	error_long_qual	PASS
**fast**	invalid	error_trunc_in_title	PASS
**fast**	invalid	error_trunc_in_plus	PASS
**fast**	invalid	error_short_qual	PASS
**fast**	invalid	error_tabs	PASS
**fast**	invalid	error_spaces	PASS
**fast**	invalid	error_double_qual	PASS
**fqtools**	valid	illumina_full_range_as_illumina	PASS
**fqtools**	valid	sanger_full_range_as_illumina	PASS
**fqtools**	valid	longreads_as_illumina	PASS
**fqtools**	valid	solexa_full_range_as_illumina	PASS
**fqtools**	valid	sanger_full_range_as_sanger	PASS
**fqtools**	valid	longreads_as_sanger	PASS
**fqtools**	valid	solexa_full_range_as_solexa	PASS
**fqtools**	valid	misc_dna_original_sanger	PASS
**fqtools**	valid	misc_dna_as_sanger	PASS
**fqtools**	valid	misc_rna_original_sanger	PASS
**fqtools**	valid	misc_rna_as_illumina	PASS
**fqtools**	valid	misc_rna_as_sanger	PASS
**fqtools**	valid	sanger_full_range_original_sanger	PASS
**fqtools**	valid	wrapping_as_solexa	PASS
**fqtools**	valid	illumina_full_range_as_sanger	PASS
**fqtools**	valid	illumina_full_range_original_illumina	PASS
**fqtools**	valid	longreads_as_solexa	PASS
**fqtools**	valid	longreads_original_sanger	PASS
**fqtools**	valid	misc_dna_as_solexa	PASS
**fqtools**	valid	solexa_full_range_original_solexa	PASS
**fqtools**	valid	misc_rna_as_solexa	PASS

**fqtools**	valid	wrapping_original_sanger	PASS
**fqtools**	valid	solexa_full_range_as_sanger	PASS
**fqtools**	valid	wrapping_as_illumina	PASS
**fqtools**	valid	wrapping_as_sanger	PASS
**fqtools**	valid	illumina_full_range_as_solexa	PASS
**fqtools**	valid	misc_dna_as_illumina	PASS
**fqtools**	valid	sanger_full_range_as_solexa	PASS
**fqtools**	invalid	error_qual_del	PASS
**fqtools**	invalid	error_diff_ids	PASS
**fqtools**	invalid	error_qual_null	PASS
**fqtools**	invalid	error_trunc_at_seq	PASS
**fqtools**	invalid	error_qual_unit_sep	PASS
**fqtools**	invalid	error_double_seq	PASS
**fqtools**	invalid	error_trunc_at_qual	PASS
**fqtools**	invalid	error_trunc_in_qual	PASS
**fqtools**	invalid	error_qual_escape	PASS
**fqtools**	invalid	error_trunc_at_plus	PASS
**fqtools**	invalid	error_no_qual	PASS
**fqtools**	invalid	error_qual_tab	PASS
**fqtools**	invalid	error_trunc_in_seq	PASS
**fqtools**	invalid	error_qual_vtab	PASS
**fqtools**	invalid	error_qual_space	PASS
**fqtools**	invalid	error_long_qual	PASS
**fqtools**	invalid	error_trunc_in_title	PASS
**fqtools**	invalid	error_trunc_in_plus	PASS
**fqtools**	invalid	error_short_qual	PASS
**fqtools**	invalid	error_tabs	PASS
**fqtools**	invalid	error_spaces	PASS
**fqtools**	invalid	error_double_qual	PASS
